# Renal cell carcinoma histologic subtypes exhibit distinct transcriptional profiles

**DOI:** 10.1172/JCI178915

**Published:** 2024-04-23

**Authors:** Pedro Barata, Shuchi Gulati, Andrew Elliott, Hans J. Hammers, Earle Burgess, Benjamin A. Gartrell, Sourat Darabi, Mehmet A. Bilen, Arnab Basu, Daniel M. Geynisman, Nancy A. Dawson, Matthew R. Zibelman, Tian Zhang, Shuanzeng Wei, Charles J. Ryan, Elisabeth I. Heath, Kelsey A. Poorman, Chadi Nabhan, Rana R. McKay

**Affiliations:** 1 Tulane Medical School, New Orleans, Louisiana, USA.; 2 University Hospitals Seidman Cancer Center, Cleveland, Ohio, USA.; 3 UC Davis Health System, Sacramento, California, USA.; 4 Caris Life Sciences, Irving, Texas, USA.; 5 Kidney Cancer Program, UT Southwestern Medical Center, Dallas, Texas, USA.; 6 Levine Cancer Institute Atrium Health, Charlotte, North Carolina, USA.; 7 Montefiore Medical Center/Albert Einstein College of Medicine, Bronx, New York, USA.; 8 Hoag Memorial Presbyterian Hospital, Newport Beach, California, USA.; 9 Winship Cancer Institute, Emory University, Atlanta, Georgia, USA.; 10 University of Alabama at Birmingham, Birmingham, Alabama, USA.; 11 Fox Chase Cancer Center, Philadelphia, Pennsylvania, USA.; 12 Georgetown University Lombardi Comprehensive Cancer Center, Washington, DC, USA.; 13 University of Minnesota, Minneapolis, Minnesota, USA.; 14 Barbara Ann Karmanos Cancer Institute, Wayne State University, Detroit, Michigan, USA.; 15 Moores Cancer Center, UCSD, San Diego, California, USA.

**Keywords:** Genetics, Oncology, Cancer, Molecular genetics, Urology

## Abstract

Molecular profiling of clear cell renal cell carcinoma (ccRCC) tumors of patients in a clinical trial has identified distinct transcriptomic signatures with predictive value, yet data in non–clear cell variants (nccRCC) are lacking. We examined the transcriptional profiles of RCC tumors representing key molecular pathways, from a multi-institutional, real-world patient cohort, including ccRCC and centrally reviewed nccRCC samples. ccRCC had increased angiogenesis signature scores compared with the heterogeneous group of nccRCC tumors, while cell cycle, fatty acid oxidation/AMPK signaling, and fatty acid synthesis/pentose phosphate signature scores were increased in one or more nccRCC subtypes. Among both ccRCC and nccRCC tumors, T effector scores statistically correlated with increased immune cell infiltration and were more commonly associated with immunotherapy-related markers (PD-L1^+^/TMB^hi^/MSI^hi^). In conclusion, this study provides evidence of differential gene transcriptional profiles among ccRCC versus nccRCC tumors, providing insights for optimizing personalized and histology-specific therapeutic strategies for patients with advanced RCC.

## Abstract 2

Renal cell carcinoma histologic subtypes have distinct expression of gene sets representing key molecular pathways with potential to personalize treatments for patients.

## Graphical abstract

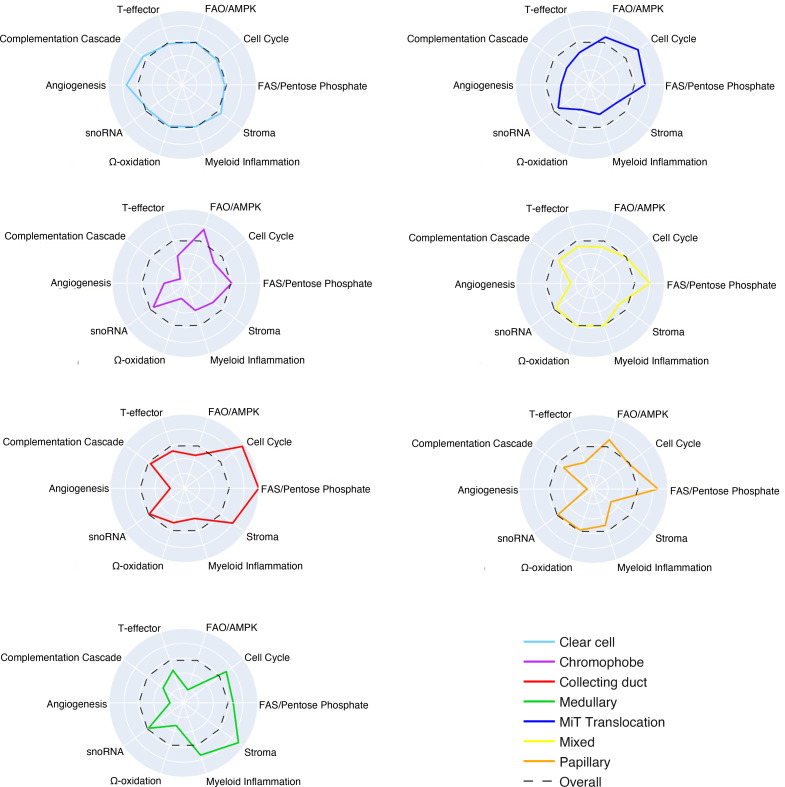

## Introduction

Renal cell carcinoma (RCC) is a common cancer among men and women in the United States, with an estimated 81,800 new cases and 14,890 deaths expected in 2023 (1). Clear cell RCC (ccRCC) is the most common subtype, representing 70%–80% of all RCCs (2, 3). Other variant histologies, which have been historically lumped together as non-ccRCC (nccRCC), have distinct clinical features and pathogenesis, including papillary, chromophobe, medullary, collecting duct, microphthalmia (MiT) family translocation, succinate dehydrogenase–deficient, hereditary leiomyomatosis and syndrome-associated, and unclassified RCC (3). Across all RCC histologies, 15%–20% harbor sarcomatoid dedifferentiation, (4) which portends poor prognosis, increased likelihood of presenting with advanced stage, and worse survival across all stages (5).

Over the past decade, the medical management of advanced RCC has significantly changed with the emergence of the immune checkpoint inhibitors and next-generation tyrosine kinase inhibitors (TKIs). Currently, front-line treatment options include combined immuno-oncology (IO-IO) or IO-TKI–based treatment (6–9). VEGF TKIs continue to be relevant and efficacious, either as monotherapy or in combination with immunotherapy (10). Tumors with rhabdoid/sarcomatoid dedifferentiation are associated with improvement in clinical outcomes, including overall response rate and progression-free survival (PFS) with IO-based approaches (11–14).

While we have made great strides in improving survival for patients with RCC in the modern era, outcomes to therapy are heterogeneous, with a subset of patients demonstrating long-term durability, while others demonstrate intrinsic resistance to treatment (6, 8, 9, 15). Most importantly, to date, there are no clinically applicable predictive biomarkers to help optimize therapy selection in the clinic. Common markers of response to immune checkpoint inhibitors, such as programmed cell death ligand 1 (PDL1) expression and tumor mutation burden (TMB) are at times associated with higher responses, yet they have not been applied clinically, given the presence of observed responses in the absence of these markers (16–18).

Important work has been done to identify transcriptomic signatures in both localized and metastatic ccRCC. Particularly, in metastatic ccRCC, gene expression signatures have been described based on markers of angiogenesis and those of immune activation. The phase II IMmotion 150 trial evaluated the clinical relevance of T effector/IFN-γ (Teff) and angiogenesis^hi/lo^ gene expression signatures identified by RNA sequencing (16). Herein, the high Teff^hi^ signature was associated with longer PFS in the atezolizumab plus bevacizumab group versus the sunitinib group. By contrast, a high angiogenic signature was associated with improved PFS in the sunitinib group. Subsequently, the randomized, global phase III IMmotion 151 integrated multi-omic analyses leading to identification of robust molecular clusters derived from analyses of 823 tumors from patients with advanced RCC, including 134 tumors with sarcomatoid features (17). A total of 7 gene clusters were identified by nonnegative matrix factorization, including inflammatory and angiogenic signatures. Cluster 1 and 2 were characterized by angiogenic genes (enriched for vascular and VEGF pathway–related genes); clusters 4, 5, and 7 showed increased expression of inflammatory pathways; and clusters 3 and 6 were characterized by high myeloid and low T effector gene expression patterns. Differential outcomes to therapy were observed in each of the clusters, which allowed us to begin to shed light on the potential clinical applicability of a biomarker selection strategy utilizing the cluster classification.

Other phase III trials, such as Javelin Renal 101 and CheckMate 214 (NCT02231749), also investigated the predictive value of transcriptomic signatures. Using a different methodology (Javelin Renal 101 [NCT02684006], a novel 26-gene expression signature derived from 720 tumors from patients enrolled in the Javelin Renal 101 trial was associated with PFS after treatment with axitinib plus avelumab versus sunitinib (18). In the exploratory analysis of CheckMate 214, including 213 samples (20% of total study cohort), the immune-based signatures, whose scores were derived from 3 IMmotion150 signatures, the Javelin Renal 101 signature, and the tumor inflammation signature, were associated with PFS in patients treated with immune checkpoint inhibitors but they failed to show an association with overall survival (Checkmate 214). Additionally, the association between angiogenic gene expression and anti-VEGF therapies was also not statistically significant (19).

Data on gene expression signatures and other molecular characterization in different RCC histologies beyond ccRCC are lacking. Here, we present data from an international, multi-institutional, real-world cohort of patients with RCC who have undergone comprehensive molecular evaluation. We aim to describe the gene expression signatures, mutational profiles, and protein expression patterns across the different RCC histologies, including tumors with sarcomatoid/rhabdoid features and non–clear cell pathologies.

## Results

### Study cohort and patient characteristics.

**Figure 1:**
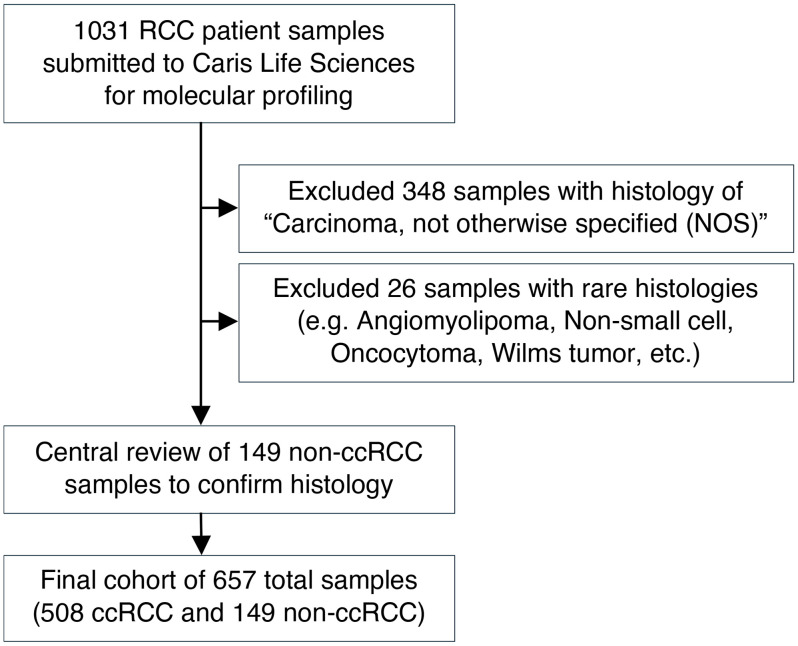
Consort diagram of study inclusion process.

The study cohort comprised a total of 657 patient samples, including both ccRCC (*n =* 508) and nccRCC (*n =* 149) histologic subtypes (Table 1 and Figure 1). Sarcomatoid and/or rhabdoid features were present in 9.4% of the overall cohort, with a significantly higher frequency in patients with nccRCC (14.1% vs. 8.1% ccRCC, *P =* 0.03), specifically in chromophobe (20.0% vs. 8.1%, *P =* 0.03) and mixed subtypes (23.5% vs. 8.1%; *P <* 0.01). Papillary RCC tumors were associated with an increased median age at the time of biopsy compared with ccRCC tumors, while medullary RCC was associated with a younger median age. MiT translocation RCC was more frequent among women (87.5% vs. 30.1% ccRCC; *P <* 0.01). Distributions of age, sex, and tissue specimen source (*n =* 337 collected from primary site, and *n =* 322 from metastatic site) were similar between ccRCC and nccRCC subtypes.

### Transcriptional characterization and stratification of samples from patients with RCC into molecular subgroups.

**Figure 2:**
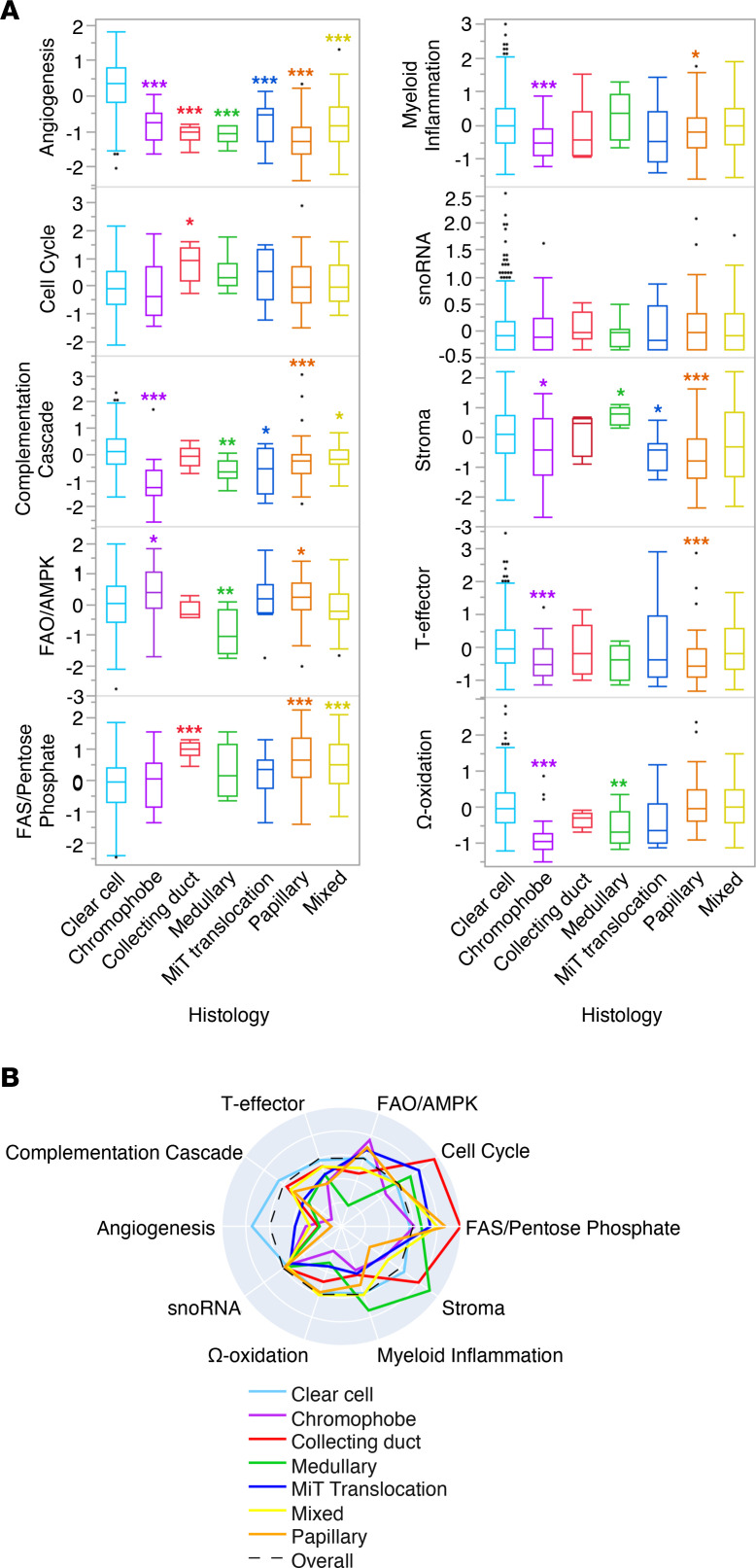
RCC subtypes exhibit distinct gene expression profiles. (**A**) Differential expression of 10 gene sets representing key molecular pathways by RCC subtype. (**B**) Radial plots of the median gene signature expression level by RCC subtype. Mann-Whitney *U* test, *P < 0.05, **P < 0.01, ***P < 0.001 when compared with ccRCC.

Prior studies of RCC have described molecular subgroups with gene expression signatures that reflect activation of key molecular pathways, including T effector (comprising *CD274*, *CD8A*, *EOMES*, *IFNG*, and *PRF1*) and angiogenic (comprising *ANGPTL4*, *CD34*, *ESM1*, *KDR*, *KDR*, *PECAM1*, and *VEGFA*) gene sets, and these subgroups were further associated with differential outcomes to therapy (17, 20). We performed gene expression profiling of 10 signatures in a cohort of real-world RCC tumor samples and characterized signature scores by histologic subtype (Figure 2).

Angiogenesis signature scores were significantly higher in ccRCC compared with all nccRCC subtypes (mean *Z* score, 0.37 vs. –0.99; *P <* 0.001), and the highest median expression of complement cascade (mean *Z* score, 0.13 vs. –0.44; *P <* 0.001) and T effector signature scores (mean *Z* score, 0.08 vs. –0.27; *P <* 0.001) was observed in ccRCC (Figure 2). Chromophobe RCC had increased fatty acid oxidation (FAO)/AMPK signaling scores (mean *Z* score, 0.38 vs. –0.02 in ccRCC; *P <* 0.05). Stromal scores were increased in medullary RCC (mean *Z* score, 0.74 vs. 0.11 in ccRCC; *P <* 0.05), with decreased scores observed for multiple signatures in both subtypes. MiT translocation RCC had increased angiogenesis with decreased complement cascade (mean *Z* score, –0.60 vs. 0.13 in ccRCC; *P <* 0.05) and stromal scores (mean *Z* score, –0.51 vs. 0.11 in ccRCC; *P <* 0.05). Cell cycle (mean *Z* score, 0.78 vs. –0.03 in ccRCC; *P <* 0.05) and fatty acid synthesis (FAS)/pentose phosphate scores (mean *Z* score, 0.97 vs. –0.14 in ccRCC; *P <* 0.001) were significantly increased in collecting duct carcinoma. Papillary and mixed tumors had increased FAS/pentose phosphate scores (mean *Z* score, 0.72 and 0.48, respectively; *P <* 0.001 each; Supplemental Figure 3).

We next examined gene expression signatures for associations with patient demographic features (Supplemental Figure 1). Compared with younger patients, older patients were associated with decreased myeloid inflammation (mean *Z* score, –0.15 vs. 0.01; *P <* 0.05) and stromal expression scores (mean *Z* score, –0.13. vs. 0.21; *P <* 0.001). RCC samples from female patients had increased angiogenesis (mean *Z* score, 0.24 vs. 0.05; *P <* 0.001), FAO/AMPK signaling (mean *Z* score, 0.23 vs. –0.02; *P <* 0.001), and FAS/pentose phosphate scores (mean *Z* score, 0.15 vs. –0.01; *P <* 0.05), while complement cascade (mean *Z* score, –0.09 vs. 0.03; *P <* 0.05) and Ω-oxidation scores (mean *Z* score, –0.13 vs. –0.05; *P <* 0.05) were decreased compared with those of male patients. Additionally, metastatic samples had higher cell cycle (mean *Z* score, 0.19 vs. –0.20; *P <* 0.001), FAS/pentose phosphate (mean *Z* score, 0.15 vs. –0.07; *P <* 0.01), stroma (mean *Z* score, 0.37 vs. –0.24; *P <* 0.001), myeloid inflammation (mean *Z* score, 0.03 vs. –0.20; *P <* 0.001), and complement cascade scores (mean *Z* score, 0.05 vs. –0.07; *P <* 0.001) compared with specimens collected from the kidney.

### Genomic alterations are differentially associated with molecular subgroups across RCC histologies.

**Figure 3:**
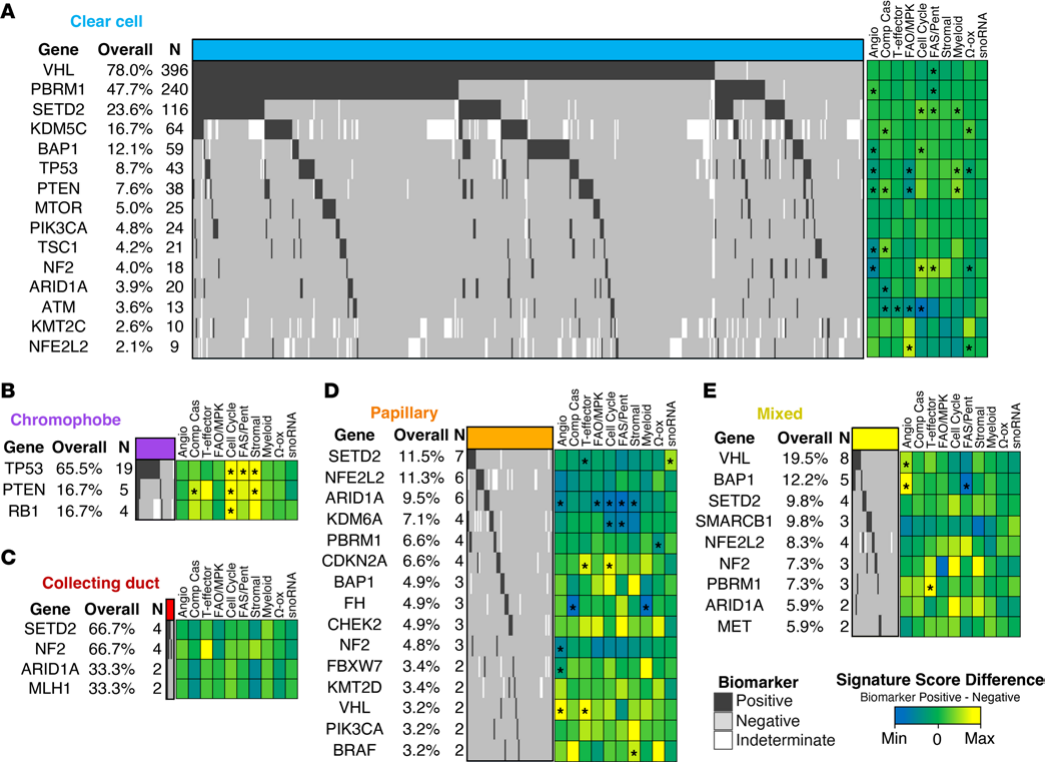
Genomic alterations associated with gene signatures across RCC histologies. Oncoprint of the most commonly altered genes, with heatmap indicating the difference in gene signature score differences between biomarker^+^ (i.e., mutated) and biomarker^–^ tumors in (**A**) clear cell, (**B**) chromophobe, (**C**) collecting duct, (**D**) papillary, and (**E**) mixed tumors. Note: genes with less than 2 altered samples were excluded. *P < 0.05, Mann-Whitney *U* test.

The most common alteration among ccRCC was *VHL (78%*, *n =* 396*)*, which was associated with lower FAS/pentose phosphate signature scores (mean *Z* score difference, –0.15 compared with *VHL* wild-type tumors; *P <* 0.05) (Figure 3A). Other commonly mutated genes among ccRCC included *PBRM1* (47.7%, *n =* 240), which associated with high angiogenesis scores (mean *Z* score difference, 0.20; *P <* 0.01) and low FAS/pentose phosphate scores (mean *Z* score difference, –0.19; *P <* 0.05); *SETD2* (23.6%, *n =* 116), which associated with cell cycle (mean *Z* score difference, 0.41; *P <* 0.001); FAS/pentose phosphate (mean *Z* score difference, 0.26; *P <* 0.05) and myeloid inflammation scores (mean *Z* score difference, 0.24; *P <* 0.01); and *KDM5C* (16.7%, *n =* 64), which associated with increased complement cascade (mean *Z* score difference, 0.31; *P <* 0.001) and Ω-oxidation signature scores (mean *Z* score difference, 0.30; *P <* 0.001). In chromophobe RCC, mutations in *TP53* (mean *Z* score difference, 1.09; *P <* 0.05), *PTEN* (mean *Z* score difference, 1.28; *P <* 0.05), and *RB1* were most prevalent and each associated with increased cell cycle scores (mean *Z* score difference, 1.42; *P <* 0.05), along with increased stromal scores in tumors with *TP53* (mean *Z* score difference, 1.48; *P <* 0.05) and *PTEN* mutations (mean *Z* score difference, 1.73; *P <* 0.05) (Figure 3B). Alterations in *SETD2*, *NF*2, *ARID1*, and *MLH1* were identified in collecting duct carcinoma samples, although none were significantly associated with gene signatures (Figure 3C). In papillary RCC, mutations in *ARID1A* (9.5%, *n =* 6) associated with decreased angiogenesis (mean *Z* score difference, –0.68; *P <* 0.01), cell cycle (mean *Z* score difference, –0.89; *P <* 0.05), FAO/AMPK signaling (mean *Z* score difference, –0.70; *P <* 0.05), FAS/pentose phosphate (mean *Z* score difference, –1.14; *P <* 0.05), and stromal scores (mean *Z* score difference, –0.75; *P <* 0.05), while *SETD2* (11.5%, *n =* 7) associated with increased snoRNA (mean *Z* score difference, 0.63; *P <* 0.05) and decreased T effector scores (mean *Z* score difference, –0.38; *P <* 0.05) (Figure 3D). In mixed tumors, mutations in *VHL* were associated with increased angiogenesis scores (mean *Z* score difference, 0.68; *P <* 0.05), while *BAP1* associated with increase angiogenesis (mean *Z* score difference, 1.08; *P <* 0.05) and decreased FAS/pentose phosphate scores (mean *Z* score difference, –1.10; *P <* 0.05) (Figure 3E).

### Molecular subgroups are associated with distinct tumor microenvironments.

**Figure 4:**
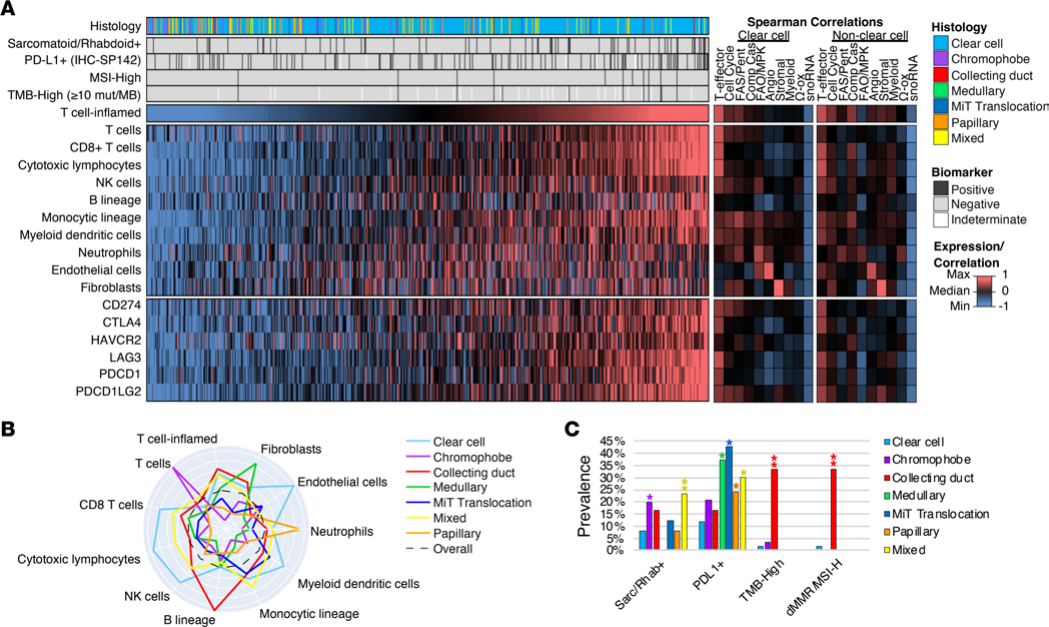
Association of gene scores with unique tumor microenvironments. (**A**) Heatmap of immuno-oncology–related (IO-related) biomarkers, relative abundance of immune and stromal cell population estimated from RNA expression, and expression of key immune checkpoint genes across all RCC samples, with adjacent heatmap indicating the Spearman’s correlation strength with gene scores. (**B**) Radial plot of the median relative abundance of cell types by RCC subtype. (**C**) Prevalence of IO-related biomarkers by RCC subtype. *P < 0.05, **P < 0.01 when compared with ccRCC, χ^2^ or Fisher’s exact test, where appropriate.

The presence of tumor-infiltrating lymphocytes predicts response to checkpoint inhibitor therapy, and we hypothesized that the gene expression profiles of molecular subgroups would be associated with differences in tumor microenvironment composition. Using the Microenvironment Cell Population–counter method (21), the relative abundance of immune and stromal populations in the tumor microenvironment was estimated from cell type–specific transcript levels. In both ccRCC and nccRCC, the T effector signature positively correlated with the presence of cytotoxic lymphocytes (Spearman’s ρ = 0.9; *P <* 0.001), T cells/CD8^+^ T cells (ρ = 0.9; *P <* 0.001), NK cells (ρ = 0.7; *P <* 0.001), monocytic lineage (ρ = 0.6; *P <* 0.001), and myeloid dendritic cell abundance (ρ = 0.6; *P <* 0.001), as well as with a “T cell–inflamed” signature that has been associated with response to immunotherapy (ρ = 0.9; *P <* 0.001) and the expression of multiple immune checkpoint genes (ρ = 0.05 to 0.8; *P <* 0.001) (Figure 4A). Endothelial cell and fibroblast abundance had the strongest association with angiogenesis (ρ = 0.9; *P <* 0.001) and stromal cell scores (ρ = 0.9; *P <* 0.001), respectively, in both ccRCC and nccRCC subtypes. Median abundance of cytotoxic lymphocytes, CD8^+^ T cells, NK cells, myeloid dendritic cells, and endothelial cells was highest in ccRCC, while B lineage, fibroblasts, neutrophils, and monocytic lineage abundance was highest in collecting duct, medullary, papillary, and mixed RCC subtypes, respectively (Figure 4B)

Sarcomatoid/rhabdoid features were present in 9.4% of the overall cohort and, compared with ccRCC (8.1%, *n =* 41), were significantly more frequent in chromophobe (20.0%, *n =* 6; *P <* 0.05) and mixed (23.5%, *n =* 8; *P <* 0.01) RCC subtypes (Figure 4C). Overall, 15.0% (*n =* 97) of RCC samples were PDL1^+^ (staining of ≥2+ intensity and ≥5% tumor cells using SP142 antibody), with significantly higher frequency of PDL1^+^ tumors in medullary (37.5%, *n =* 3; *P <* 0.05), MiT translocation (42.9%, *n =* 3; *P <* 0.05), papillary (24.2%, *n =* 14; *P <* 0.05), and mixed (26.5%, *n =* 9; *P <* 0.05) RCC compared with ccRCC (12.0%, *n =* 60; *P <* 0.05). The overall median TMB was 4 mutations/megabase, and TMB-high subtypes (≥10 mutations/megabase) were observed in 1.9% (*n =* 12) of all RCC samples, most frequently among collecting duct carcinoma (33.3%, *n =* 2, vs. ccRCC 1.8%, *n =* 9; *P <* 0.01), and often concurrent with mismatch repair deficient/microsatellite instability^hi^ (dMMR/MSI-H) status.

### Sarcomatoid/rhabdoid features are associated with unique molecular and immune profiles.

**Figure 5:**
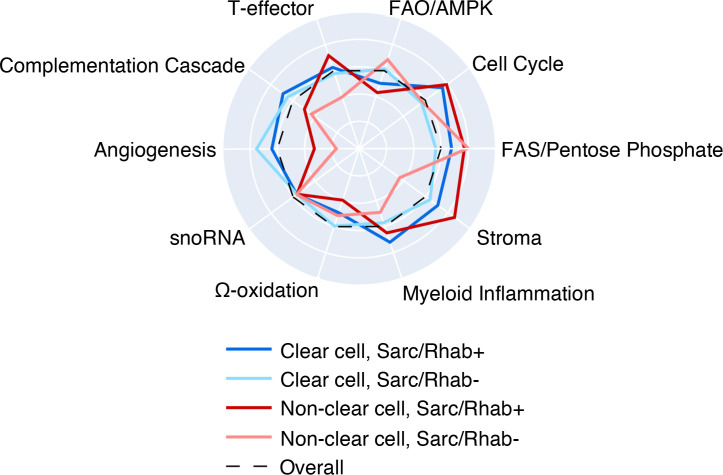
Sarcomatoid/rhabdoid features are associated with distinct expression profiles. Radial plot of the median gene signature expression level by subgroups.

The presence of sarcomatoid/rhabdoid features in both clear cell and nccRCC subtypes was associated with increased T effector, cell cycle, myeloid inflammation, and stromal signature scores as well as decreased FAO/AMPK signaling scores (Figure 5 and Table 2). Interestingly, several associations between gene alteration and signature score varied by histological subtype and the presence of sarcomatoid/rhabdoid features (Supplemental Figure 2). For example, *SETD2* mutations were associated with lower stromal scores in ccRCC with sarcomatoid/rhabdoid features (mean *Z* score difference, –0.87; *P <* 0.05) but higher stromal scores in ccRCC without sarcomatoid/rhabdoid features (mean *Z* score difference, –0.87; *P <* 0.05). However, *TP53* mutations were similarly associated with decreased complement cascade scores in nccRCC, regardless of sarcomatoid/rhabdoid features (mean *Z* score difference, –0.84 in tumors with sarcomatoid or rhabdoid features, –0.99 in tumors without sarcomatoid or rhabdoid; *P <* 0.01), in addition to increased stromal in tumors with sarcomatoid or rhabdoid features (mean *Z* score difference, 1.47; *P <* 0.05) and increased angiogenesis scores in tumors without sarcomatoid or rhabdoid (mean *Z* score difference, 0.43; *P <* 0.01).

## Discussion

Our analysis of a large cohort of real-world patient samples is concordant with that of recent trial reports on gene expression signatures in ccRCC (14, 17, 19). As data on nccRCC are sparse, our findings among a subpopulation of centrally confirmed cases of nccRCC subtypes provide valuable insights into the specific molecular pathways and immune microenvironment of each RCC subtype and their associations with other clinical markers of interest. A better understanding of the molecular underpinnings and gene expression patterns across RCC subtypes will be critical for informing therapeutic strategies for patients with variant histology RCC, a group that has historically been underrepresented in clinical trials and continues to represent an unmet need. Our comparative analyses of ccRCC and nccRCC subtypes revealed histology-specific and biomarker-associated expression of key molecular pathways to provide insights for these rare patient populations.

Clear cell samples were predominant in this study cohort, with a similar proportion of cases (77%) to real-world prevalence rates (22, 23). Concordant with other large ccRCC cohorts, such as the Cancer Genome Atlas Research Network (24), DNA-sequencing data revealed frequent alterations in genes controlling cellular oxygen sensing (e.g., *VHL*) as well as chromatin remodeling genes, such as *PBRM1*, *SETD2*, and *BAP1*. Both angiogenic and myeloid inflammation scores were higher in ccRCC tumors compared with nccRCC tumors. The most abundant immune cell types in ccRCC samples were CD8^+^ T cells, macrophages, and CD4^+^ T cells, consistent with previous reports (25). However, it has been shown that clear cell tumors are clustered into distinct molecular subgroups with different distribution of immune cells; in our analysis, the differential association of cell population with molecular subgroups seem to support such findings (25). Single-cell transcriptomic profiling of immune cells has detected a higher proportion of exhausted CD8^+^ T cell in advanced disease compared with earlier stages (26) and higher levels of coinhibitory receptors and effector molecules in cytotoxic T cells among responders to immunotherapy (27). At the somatic level, *PBRM1* mutations have been associated with a less immunogenic tumor microenvironment and upregulated angiogenesis and have suggested more limited benefit from immunotherapy (28–30). The lack of clinical annotation and integration of single-cell sequencing prevented us from confirming these findings and require further validation in future real-world data sets.

Papillary RCC was the most represented nccRCC subtype in our analysis, as expected from epidemiology studies (31). Papillary is no longer subclassified into type 1 and type 2, yet we found molecular alterations reported historically present in type 1 subtype, such as *MET* alterations, and type 2 subtype, including chromatin modification (e.g., *ARID1A*, *SETD2*), NRF2 pathway (e.g., *FH, NFE2L2*), and the Hippo pathway (e.g., *NF2*) (32). The lower angiogenic scores relative to ccRCC are concordant with the observed lower activity of anti-VEGF inhibitors in these tumors (33, 34). Furthermore, the presence of inflammatory gene scores, immune-related markers, and immune cell populations in these tumors might help explain the clinical efficacy that immune checkpoint inhibitors have shown in these tumors, either as monotherapy or in combination with anti-VEGF TKIs (35, 36).

To a lesser extent, our cohort included patients with papillary and other nccRCC subtypes, and we identified differential gene expression scores: chromophobe RCC had increased FAO/AMPK signaling scores, while stromal scores were increased in medullary RCC. Cell cycle and FAS/pentose phosphate scores were significantly increased in collecting duct carcinoma. Chromophobe RCC is known to be associated with multiple losses of chromosomes 1, 2, 6, 10, 13, 17, and 21, and *TP53* and *PTEN* are the two most frequently mutated genes. Genomic structural arrangements involving the *TERT* promoter region, as well as diffusely increased mitochondrial function and mitochondrial DNA alterations, are more common in chromophobe RCC, which was identified in our cohort as well (37, 38). Sarcomatoid/rhabdoid features were frequently found (20%) in these tumors as previously reported (39), yet immunotherapies continue to show limited activity in these tumors (35, 40). Of note, nonsarcomatoid chromophobe tumors had similar mutation frequencies of TP53 (61%), RB1 (15%), and PTEN (13%) as did tumors in the overall analysis, along with similar expression of the 10 gene sets representing key molecular pathways, with exception of the “stroma” gene set that enriched in chromophobe tumors with sarcomatoid features present (Figure 5).

Collecting duct samples, which are characterized by frequent genomic alterations involving *NF2*, *SETD2*, *ARID1A*, and *SMARCB1* (31, 41), had the highest median myeloid inflammation expression score while having one of the lowest angiogenesis scores. These findings may help to explain the clinical reports of relative success of mTOR inhibitors in the *NF2*-mutated cases as well as disease control rates with immune checkpoint inhibitors, while antiangiogenic therapies and chemotherapy are of limited value (41).

Owing to the rarity of MiT translocation, our cohort included only a limited number of molecularly confirmed cases, which had a clear female predominance and younger age at presentation, as expected (42). Angiogenesis, complement cascade, and stroma expression scores were decreased compared with ccRCC, but the lack of recurrent coalterations precluded further analysis of biomarker associations.

Finally, there was a strong association between sarcomatoid/rhabdoid^+^ tumors and high myeloid inflammation scores and low angiogenic scores. While this association has been observed in some trial reports (e.g., IMmotion151) but not others (e.g., CheckMate 214), variations in methodologies of analysis and availability of tissue samples across these studies limit cross trial comparisons of this correlative data (19, 20).

While we highlight results from a large data set of genomically profiled distinct RCC tumors, there are several limitations to this work. Limited clinical data available in the database prevented us from investigating the presence of the gene expression scores by IMDC prognostic risk groups. Similarly, the predictive value of the transcriptomic scores could not be assessed. Rarer forms of RCC, such as collecting duct, medullary, and translocation RCC, were underrepresented in this cohort and require molecular profiling of additional samples in future studies to verify results. While we presume that most samples were submitted for molecular profiling at the time of advanced disease based on clinical guidelines for molecular testing, precise staging information was not available. The effect of systemic therapies on the molecular characterization of tumors is largely unknown, and tumor clonal heterogeneity and evolution could not be assessed. Future studies in both clear cell, such as the OPTIC trial (NCT05361720), and in variant RCC subtypes that incorporate gene expression scores are required to validate their predictive value and help with patient selection.

In conclusion, despite these limitations, we were able to identify distinct transcriptional profiles across multiple RCC histologies from a large cohort of real-world samples from patients with RCC. The findings of our work are concordant with prior trial data, suggesting potential clinical significance and therapeutic implications. Future directions include independent prospective validation of these findings in the context of different systemic therapies that are currently available or under development.

## Methods

### Sex as a biological variant.

Samples from both male and female participants were involved in this research, as the findings do apply to both groups.

### Study cohort.

Clinical physicians submitted FFPE samples from patients with kidney cancer (*n =* 657) to a commercial CLIA-certified laboratory for molecular profiling (Caris Life Sciences) (Figure 1). All tumor samples categorized as variant histologies underwent central pathology review at Caris Life Sciences. Tumors classified as mixed subtypes included samples with histologic features of more than one subtype, most commonly papillary with clear cell changes or unspecific features. The MiT family translocation subtype was confirmed by tumor genomic sequencing.

### Clinical characteristics.

Limited baseline clinical factors, such as age and sex as a biological variable (male and female), were available and included in this study.

### DNA next-generation sequencing.

Next-generation sequencing was performed on isolated genomic DNA using the NextSeq platform (Illumina Inc.) for 592 cancer-relevant genes (*n =* 375 samples) or the Illumina NovaSeq 6000 platform (Illumina Inc.) for whole-exome sequencing (WES) (*n =* 282 samples). Prior to molecular testing, tumor enrichment was achieved by harvesting targeted tissue using manual microdissection techniques. A custom-designed SureSelect XT assay was used to enrich exonic regions of 592 whole-gene targets (Agilent Technologies). All variants were detected with more than 99% confidence based on allele frequency and amplicon coverage, with an average sequencing depth of coverage of more than 500 and an analytic sensitivity threshold of 5% established for variant calling. For WES, a hybrid pull-down panel of baits designed to enrich for more than 700 clinically relevant genes at high coverage and high read-depth was used, along with another panel designed to enrich for an additional >20,000 genes at lower depth, and a 500 Mb SNP backbone panel (Agilent Technologies) was added to assist with gene amplification/deletion measurements and other analyses. Genomic variants were classified by board-certified molecular geneticists according to criteria established by the American College of Medical Genetics and Genomics. When assessing mutation frequencies of individual genes, “pathogenic” and “likely pathogenic” were counted as mutations, while “benign,” “likely benign” variants, and “variants of unknown significance” were excluded.

### RNA whole-transcriptome sequencing and fusion detection.

Whole-transcriptome sequencing uses a hybrid-capture method to pull down the full transcriptome from FFPE tumor samples using the SureSelect Human All Exon V7 bait panel (Agilent Technologies) and the Illumina NovaSeq platform. FFPE specimens underwent pathology review to discern the percentage of tumor content and tumor size; a minimum of 10% tumor content in the area for microdissection was required to enable enrichment and extraction of tumor-specific RNA. The Qiagen RNA FFPE tissue extraction kit was used for extraction, and the RNA quality and quantity were determined using the Agilent TapeStation. Biotinylated RNA baits were hybridized to the synthesized and purified cDNA targets, and the bait-target complexes were amplified in a postcapture PCR reaction. The resultant libraries were quantified and normalized, and the pooled libraries were denatured, diluted, and sequenced. Raw data were demultiplexed using the Illumina DRAGEN FFPE accelerator. FASTQ files were aligned with STAR aligner (https://github.com/alexdobin/STAR/releases/tag/2.7.4a, commit ID 04a67a8; Alexander Dobin, Arc Institute, Palo Alto, California, USA; release 2.7.4a). A full 22,948-gene data set of expression data were produced by the Salmon, which provides fast and bias-aware quantification of transcript expression (43). BAM files from STAR aligner were further processed for RNA variants using a proprietary custom detection pipeline. The reference genome used was GRCh37/hg19, and analytical validation of this test demonstrated ≥97% positive percent agreement, ≥99% negative percent agreement, and ≥99% overall percent agreement with a validated comparator method. Identified fusion transcripts were further evaluated to determine breakpoint positions and functional domains retained from fused genes.

### RNA expression analyses.

Previously described gene sets that represent key molecular pathways among transcriptionally distinct RCC subpopulations were evaluated (17). Gene expression values were log-transformed and standardized to *Z* scores, with a composite signature score calculated as the mean *Z* score of the gene set for each sample.

To assess the relative abundance of immune and stromal cell populations in the tumor microenvironment, gene expression values were analyzed using the Microenvironment Cell Populations–counter tool (21).

### Immunohistochemistry.

Immunohistochemistry was performed on full FFPE sections of glass slides. Slides were stained using the Agilent DAKO Link 48 automated platform and staining techniques, per the manufacturer’s instructions, and were optimized and validated per CLIA/CAP and ISO requirements. Staining was scored for intensity (0, no staining; 1+, weak staining; 2+, moderate staining; 3+, strong staining) and staining percentage (0%–100%). PDL1 (SP142) staining results were categorized as positive (≥2+ and ≥5% tumor cells) or negative (0 or 0%).

### Tumor mutational burden.

Tumor mutational burden was measured by counting all nonsynonymous missense, nonsense, in-frame insertion/deletion, and frameshift mutations found per tumor that had not been previously described as germline alterations in dbSNP151 (https://genome.ucsc.edu/cgi-bin/hgTrackUi?db=hg38&g=snp151), Genome Aggregation Database (gnomAD; https://gnomad.broadinstitute.org) database, or benign variants identified by Caris’s geneticists. A cutoff point of ≥10 mutations per megabase (mt/MB) was used based on the KEYNOTE-158 pembrolizumab trial (44).

### Patient summary.

Renal cell carcinoma histologic subtypes have distinct expression of gene sets representing key molecular pathways with potential to personalize treatments for patients.

### Statistics.

All statistical analyses were performed with JMP V13.2.1 (SAS Institute) or R (version 3.6.1; https://www.R-project.org). Continuous data were assessed using Mann-Whitney *U* test, and categorical data were evaluated using χ^2^ or Fisher’s exact test, where appropriate. *P* values of less than 0.05 were considered significant.

### Study approval.

The present study was conducted in accordance with the guidelines of the Declaration of Helsinki, Belmont Report, and US Common Rule. With compliance to policy 45 CFR 46.101(b), this study was conducted using retrospective, deidentified clinical data, and patient consent was not required.

### Data availability.

The data sets generated during and/or analyzed during the current study (including the figures in the manuscript and supplemental materials) are available from the corresponding author on reasonable request. The deidentified sequencing data are owned by Caris Life Sciences, and qualified researchers can apply for access to these summarized data by contacting AE and signing a data usage agreement. Values for all data points in graphs are reported in the Supporting Data Values file.

## Author contributions

PB, AE, and RRM contributed to the initial design of this study. PB, SG, AE, and RRM contributed to the development of this study, data acquisition, and writing the manuscript. PB, SG, AE, HJH, EB, BAG, SD, MAB, AB, DMG, NAD, MRZ, TZ, SW, CJR, EIH, KAP, CN, and RRM were involved in the analysis of the data and writing the final version of the manuscript. PB is listed as the first co–first author based on PB’s contribution to this project.

## Supplementary Material

Supplemental data

Supporting data values

## Figures and Tables

**Table 2:**
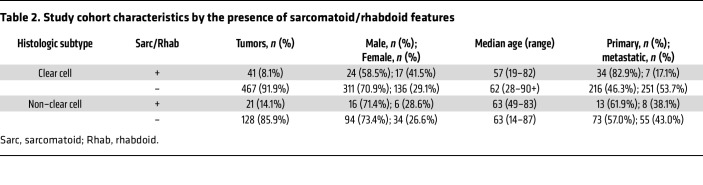
Study cohort characteristics by the presence of sarcomatoid/rhabdoid features

**Table 1:**
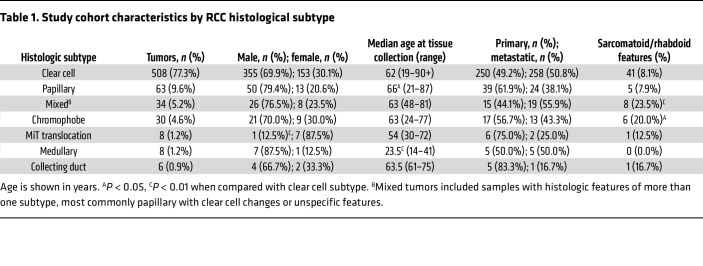
Study cohort characteristics by RCC histological subtype

## Version 1 04/23/2024

In-Press Preview

## Version 2 06/03/2024

Electronic publication
