# Supplementary material for: Renal cell carcinoma histologic subtypes exhibit distinct transcriptional profiles
Source: J Clin Invest. 2024 Apr 23;134(11):e178915. doi: 10.1172/JCI178915 (PMC11142736; doi:10.1172/JCI178915)

Supplemental Figure S1 - Radial plots of the median gene signature expression level by patient demographics.

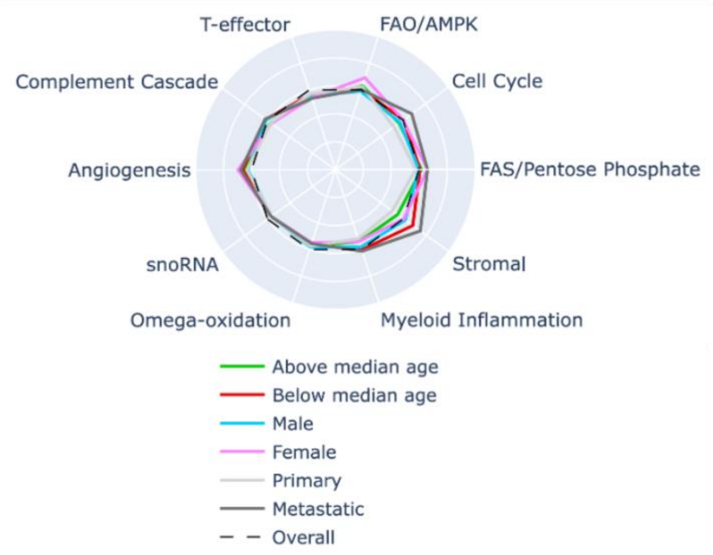

Supplemental Figure S2 - Heatmap of gene signature score differences between biomarker-positive (i.e. mutated) and -negative tumors. Note: Genes with < 2 altered samples were excluded. Mann-Whitney U test: *P<0.05.

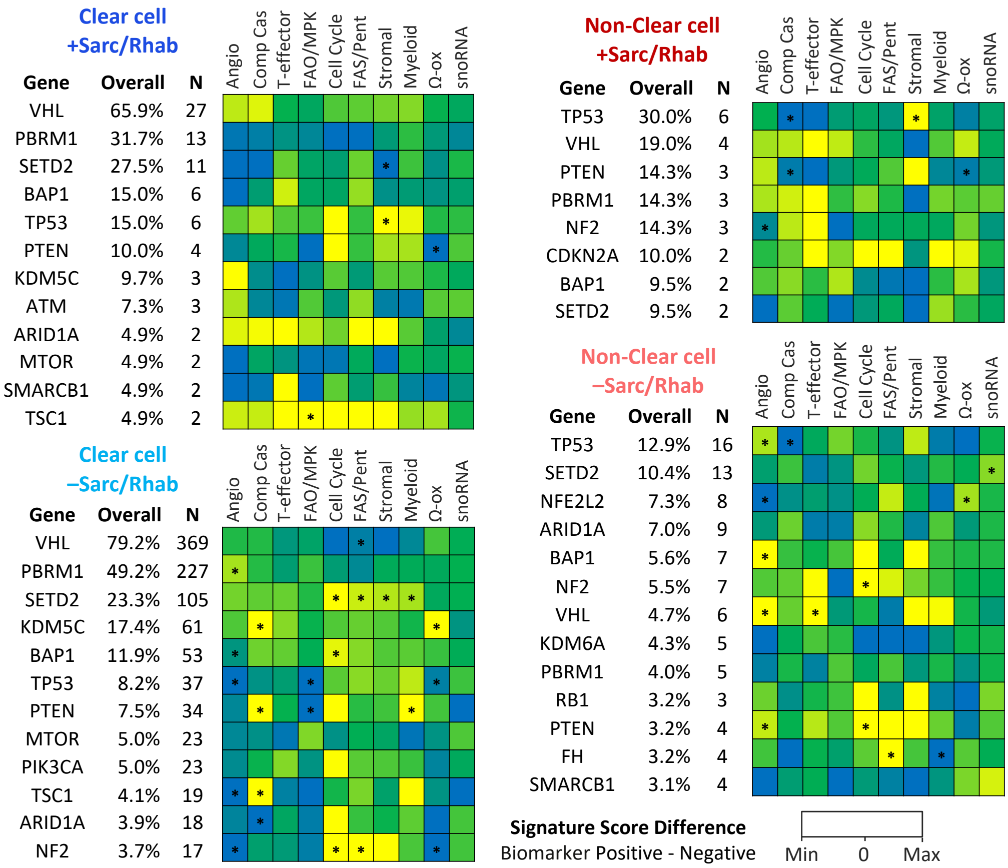

Supplemental Figure S3 - Radial plots of the median gene signature expression level for each RCC subtype, including clear cell (A), chromophobe (B), collecting duct (C), medullary (D), MiT family translocation (E), mixed (F), and papillary (G). Black dotted line represents the overall study cohort median expression level.

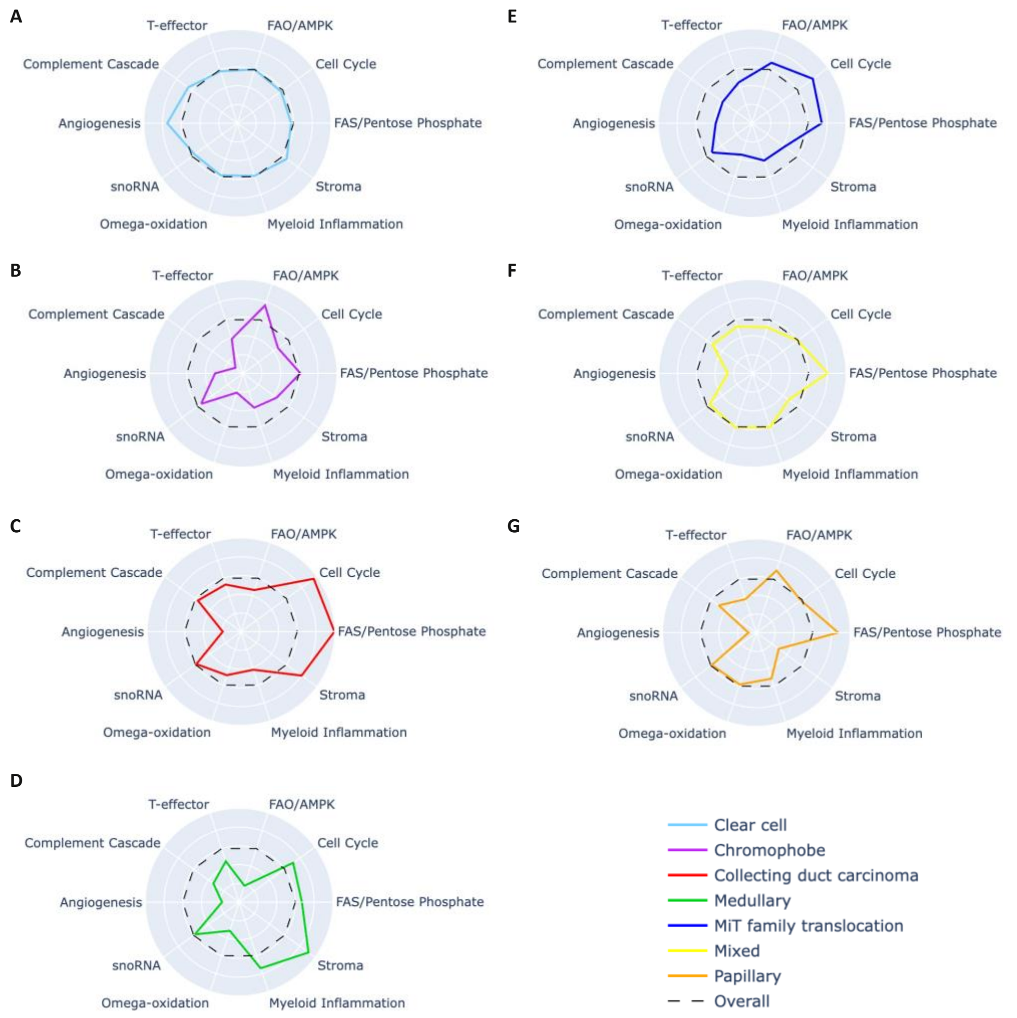

Supplement: Supplemental data [file jci-134-178915-s136.pdf]
